# Design of Experiment Approach for Enhancing the Dissolution Profile and Robustness of Loratadine Tablet Using D-α-Tocopheryl Polyethylene Glycol 1000 Succinate

**DOI:** 10.3390/pharmaceutics17030380

**Published:** 2025-03-17

**Authors:** Alhasan A. Jabbar, Israa Al-Ani, Ramadan I. Al-Shdefat, Nadia Ghazal, Anwar Jaffal, Mohamed H. Fayed

**Affiliations:** 1 Faculty of Pharmacy, Al-Ahliyya Amman University, Amman 19328, Jordan; alhasanali050@gmail.com (A.A.J.); ialani@ammanu.edu.jo (I.A.-A.); janwwar@yahoo.com (A.J.); 2 Faculty of Pharmacy, Jadara University, Irbid 21110, Jordan; rshdefat@jadara.edu.jo (R.I.A.-S.); n.ghazal@jadara.edu.jo (N.G.); 3 Department of Pharmaceutics, College of Pharmacy, University of Hafr Albatin, Hafr Albatin 1991, Saudi Arabia; 4 Department of Pharmaceutics, Faculty of Pharmacy, Fayoum University, Fayoum 63514, Egypt

**Keywords:** design of experiment, TPGS, loratadine, dissolution, tablet

## Abstract

**Background:** Formulating poorly water-soluble drugs poses significant challenges due to their limited solubility and bioavailability. Loratadine (LTD), classified as a BCS II molecule, exhibits notably low solubility, leading to reduced bioavailability. Objective: This study aims to enhance the dissolution rate of LTD through the utilization of the wet granulation process using Tocopheryl polyethylene glycol 1000 succinate (TPGS). **Methods**: A Design-of-Experiment methodology was adopted to investigate and optimize the formulation variables for preparing an oral delivery system of LTD with improved dissolution properties. The levels of TPGS (2–6% *w*/*w*), as a surfactant, and sodium starch glycolate (SSG; 2–8% *w*/*w*), as a super-disintegrant, were established as independent variables in the formulations. Loratadine was granulated in the presence of TPGS, and the resultant granules were subsequently compressed into tablets. The granules and tablets produced were then subjected to characterization. **Results**: ANOVA analysis indicated that both TPGS and SSG had a significant (*p* < 0.05) influence on the critical characteristics of the obtained granules and tablets, with TPGS showing a particularly notable effect. The optimal concentrations of TPGS and SSG for the development of LTD tablets with the necessary quality attributes were identified as 5.0% *w*/*w* and 2.0% *w*/*w*, respectively, through optimization utilizing the desirability function. The tablets produced at these optimized concentrations displayed favorable properties concerning their mechanical strength (5.72 ± 0.32 KP), disintegration time (7.11 ± 1.08 min.), and release profile (86.21 ± 1.61%). **Conclusions**: In conclusion, incorporating TPGS in the granulation process shows promise in improving the dissolution profile of poorly water-soluble drugs and demonstrated formulation robustness.

## 1. Introduction

The pharmaceutical industry is currently facing a critical challenge with the growing prevalence of poorly water-soluble drug candidates [1]. Numerous drugs have limited clinical applications because of their poor aqueous solubility, resulting in poor oral bioavailability and significant variability among individuals [2,3]. Throughout the drug discovery and development phases, the development of oral dosage forms of poorly water-soluble drugs has proven to be a challenging task in achieving optimal systemic exposure and effectiveness [4].

The sluggish rate of dissolution stands out as a major factor constraining the bioavailability of drugs classified under the Biopharmaceutical Classification System (BCS) II, characterized by favorable membrane permeability but limited solubility in water. Improving the dissolution of these drugs, known for their gastrointestinal absorption limited by their dissolution rate, typically results in increased bioavailability [5]. Various formulation strategies have been implemented to address the formulation obstacles associated with poorly water-soluble drugs. These approaches include techniques such as drug micronization, salt formation, nanosuspensions, solid dispersion, and the use of surfactants [3,5,6,7]. Historically, surfactants have been utilized to improve the solubility and bioavailability of lipophilic compounds [8,9].

The esterification of vitamin E succinate with polyethylene glycol results in the creation of a D-alpha-tocopheryl polyethylene glycol 1000 succinate (TPGS) surfactant that is characterized by non-ionic and amphiphilic properties [10]. The TPGS has garnered considerable attention across diverse drug delivery applications for its role in enhancing the bioavailability of poorly water-soluble compounds. This is achieved by improving dissolution, solubility, absorption, and permeation [11]. Typically, non-ionic surfactants like TPGS are favored over ionic surfactants due to their superior safety and compatibility profiles. Despite being arguably less studied in the context of solid oral tablets, TPGS stands out as a promising option for the formulation design, given its favorable physicochemical properties [4].

The amphiphilic nature of TPGS makes it a promising solubilizer, stabilizer, and emulsifier. It comprises a hydrophilic PEG moiety and a lipophilic vitamin E moiety, along with substantial surface activity. This quality renders TPGS effective for various poorly water-soluble drugs. It is widely used not only in immediate-release systems, but also in sustained, controlled, and targeted drug delivery applications [4,10]. Unlike other solubilizing agents, TPGS offers unique advantages, including its ability to act as both a solubilizer and a permeation enhancer, thereby improving drug absorption [12]. Additionally, TPGS stabilizes drug molecules in the gastrointestinal tract and enhances their bioavailability by inhibiting the P-glycoprotein efflux [12]. These properties make TPGS a superior choice for formulating poorly water-soluble drugs compared to traditional solubilizing agents. 

Jae-Young Lee et al. demonstrated that Soluplus^®^- and TPGS-based solid dispersions, prepared by the hot-melt extrusion process, significantly improved the aqueous solubility, dissolution rate, and oral absorption of poorly water-soluble drugs [13]. Similarly, Javier and co-workers developed solid dispersions incorporating TPGS to enhance the oral bioavailability of carvedilol, achieving a 2.1-fold increase in the solubility and dissolution rate, along with a 1.4-fold increase in AUC compared to plain carvedilol [14]. Furthermore, Sotthivirat et al. successfully incorporated TPGS into dual-compound formulations using high-shear wet granulation. This approach enhanced the dissolution and bioavailability of MK-8408, but also improved downstream processing by enhancing the flowability and reducing segregation risks [11]. These studies underscore the versatility and effectiveness of TPGS in oral solid formulations, supporting its application in our study to improve the dissolution properties of LTD.

While TPGS offers notable advantages in pharmaceutical applications, there is a paucity of references documenting its utilization in conventional oral solid formulations. The limited literature indicates that the inclusion of TPGS in these formulations can result in challenges related to tablet robustness [8]. There are no existing studies on the tableting characteristics of Loratadine (LTD) combined with TPGS. To the best of our knowledge, this study represents the inaugural exploration of this domain. Therefore, the objective of this study was to present a systematic approach for creating solid oral formulations incorporating TPGS, aiming to enhance the dissolution of LTD without compromising the tablet integrity. Tablet integrity is crucial as it ensures the physical stability, mechanical strength, and consistent drug release of the dosage form. Compromised integrity can lead to defects such as cracking, chipping, or poor dissolution, which may affect patient compliance and therapeutic efficacy [15]. Through the present method, we highlighted that incorporating TPGS in a conventional wet granulation process can enhance the dissolution of LTD without causing any tablet defects.

Loratadine is commonly prescribed as an anti-histamine agent. It is effective in treating allergic disorders with minimal side effects [16]. Allergic disorders result from hypersensitivity reactions to environmental allergens, mediated by a histamine release. Histamine activates H1 receptors, causing symptoms like vasodilation, bronchoconstriction, and gastrointestinal spasms, which manifest as rhinitis, itching, and hives [17]. Loratadine blocks H1 receptors, alleviating these symptoms. Immediate-release loratadine formulations are crucial for rapid symptom relief due to their quick dissolution and onset of action, making them ideal for patients needing prompt treatment [17].

Classified as a class II drug according to the BCS, LTD is characterized by low water solubility (water solubility of 0.004 mg/mL) and high permeability. Further, the solubility of LTD poses a significant challenge for pharmaceutical developers, aiming to enhance its bioavailability. LTD’s oral bioavailability is less than 40% primarily due to substantial first-pass metabolism [2]. Loratadine undergoes rapid first-pass metabolism in the liver, primarily mediated by cytochrome P450 enzymes, including CYP3A4 and CYP2D6. It is metabolized to its active form, desloratadine (des-carboethoxy loratadine), which is approximately four times more potent than loratadine itself [17]. Various techniques have been investigated to improve the solubility of LTD, including micellar solubilization, solid dispersion, and the formation of inclusion complexes with β-cyclodextrin [2,16]. These approaches often come with drawbacks, such as limited bioavailability and an occasional occurrence of toxic side effects [18].

To attain a delivery system with the desired quality, it is crucial to consider formulation variables. Variability in formulation parameters may lead to the failure of product quality [19]. Therefore, the Quality-by-Design (QbD) principle, serving as an evidence-based approach for developing a product with desired attributes [20,21], was implemented in the present study. To implement the QbD approach, it is strongly recommended to use the Design of Experiments (DoE) [20,22]. As a component of the QbD approach, the DoE enables the creation of a design space, providing a quantitative definition of the relationship between independent variables (i.e., formulation variables) and dependent responses. This is imperative to guarantee the quality of pharmaceutical products, drug formulations, and ultimately, patient safety [22].

## 2. Materials and Methods

### 2.1. Materials

Loratadine (LTD), Microcrystalline cellulose (MCC), and Lactose monohydrate were kindly donated by MS Pharma Co., Amman, Jordan. D-alpha-tocopheryl polyethylene glycol 1000 succinate (TPGS) was procured from Wuhan Healthdream Biological Technology Co., Ltd., Hubei, China. Sodium Starch Glycolate (SSG) and Magnesium Stearate were gifted from Dar AlDawaa Co., Amman, Jordan. All other chemicals were of analytical grades.

### 2.2. Design of Experiment and Statistical Data Analysis

The Design of Experiment (DoE) stands out as a potent statistical technique utilized for variable screening and optimization. Its effectiveness lies in the simultaneous variation of multiple factors, aiming to identify the parameter configuration that optimizes one or more desired outputs. The key advantage is its ability to achieve this optimization while minimizing the number of experimental runs required for testing, resulting in a highly cost-effective and time-efficient process [23]. To enhance the dissolution behavior of poorly water-soluble LTD, a two-factor, three-level (3^2^), full-factorial statistical design was employed. The formulation variables, TPGS (X_1_; 2–6% *w*/*w*) and SSG (X_2_; 2–8% *w*/*w*), were selected as independent variables for this investigation based on preliminary experiments and the prior literature. Previous studies have demonstrated that TPGS concentrations within this range effectively enhance drug solubility and dissolution without compromising the tablet integrity [4,11]. Similarly, SSG levels of 2–8% *w*/*w* have been shown to optimize tablet disintegration and drug release [24]. These ranges were further validated through preliminary experiments, which indicated that they provide a suitable balance between the drug release performance and tablet mechanical properties. The impact of these independent variables on the dependent responses (Y_1_: hardness, Y_2_: disintegration time, and Y_3_: dissolution behavior) was analyzed using Design Expert^®^ 12 software (version 12, State-ease, Inc., Minneapolis, MN, USA). The software generated nine experiments with three repeated center points. To enhance the predictability of the model, the experiments were conducted in a random order. Table 1 presents the independent factors and their designated levels in this study.

An ANOVA analysis, conducted at a significance level of *p* < 0.05, aimed to identify the significant effects of variables on the chosen responses. The regression model interpretation for two quantitative variables can be succinctly stated as follows (Equation (1)):

(1) $$y=\alpha_{0}+\alpha_{1}X_{1}+\alpha_{2}X_{2}+\alpha_{12}X_{1}X_{2}+\alpha_{11}{X_{1}}^{2}+\alpha_{22}{X_{2}}^{2}$$

In the context of this equation, where y represents the response variable, α is the effect coefficient specific to a factor that needs calculation, X_1_ and X_2_ are levels of the first and second factors respectively, and α_0_ stands for the intercept, which is a constant term. The validity of the design was confirmed by calculating the prediction error (PE) using Equation (2). Further, a PE of less than 5% affirms the accuracy and robustness of the selected models [25].

(2) $$PE=100\times \left(\frac{Predicted\;value-Experimental\;value}{Predicted\;value}\right)$$

### 2.3. Preparation of Granules and Tablets

Table 2 details the composition of the LTD formulations. To prepare the granulating liquid, a specified amount of TPGS was dissolved in 30 mL of hydroalcoholic solution (15% *v*/*v* alcohol and 85% *v/v* water) and then diluted to 210 mL. In a v-shaped blender (Erweka, Apparatebau, Germany), the corresponding amounts of LTD and MCC were mixed for 5 min at 50 rpm. The SSG was added to the mixture and further mixed for an additional 5 min. The blend was transferred to a mortar and wet massed by gradually adding the granulating liquid. The resulting wet mass was sieved through an 850 µm sieve to produce granules. These granules were dried in a humidity-controlled oven at (70−75 °C) for (1.75–2 h) to achieve a moisture content of (1−3%). Subsequently, the granules were sieved using 350 µm and 250 µm sieves for homogenization. The granules that passed the 350 µm sieve but adhered to the 250 µm sieve were considered successful.

The dried granules (200 g) were blended with 1% magnesium stearate for an additional 2 min at 40 rpm. The lubricated blend was then removed from the mixer and loaded into the hopper of the tablet press (KILIAN^®^, Single tip-B-Type, Berlin, Germany) equipped with an 8.4 mm diameter, and subsequently compressed into 200 mg tablets at a compression force of 10 KN based on preliminary robustness studies. This force has been shown to produce tablets with optimal mechanical strength and disintegration properties, ensuring adequate hardness without compromising the drug release performance. The resulting tablets were gathered and preserved in a securely sealed glass container for subsequent characterization.

### 2.4. Evaluation of Granules

The physical attributes of the granules were assessed in accordance with the procedures outlined in the United States Pharmacopeia (USP) [26]. Various parameters such as granule size distribution, flowability, and compressibility were examined to ensure optimal tablet compression.

#### 2.4.1. Granules Size Distribution

To verify that the formulated granules exhibited a favorable size distribution, 50 g of the granules underwent sieving using sieves with pore sizes ranging from 250 µm to 850 µm through a sieve shaker for a duration of 5 min. The sieves used for homogenization were specifically 250 µm and 350 µm, ensuring that the granules fell within the desired range for further processing. The sieves’ weights were meticulously measured before and after the sieving process to ascertain the quantity of granules retained by each sieve. 

#### 2.4.2. Flowability of Granules

The angle of repose serves as a simple yet effective method for evaluating the flow properties of a powder. In this investigation, a funnel with a 10 cm diameter and a 1.2 cm orifice was utilized. A quantity of 50 g of granules was introduced into the funnel, and the distance between the flat surface and the lower orifice was adjusted to fall within the range of 2−4 cm. The dimensions of the resulting cone, including its height (H) and diameter (D), were measured using a ruler. The angle of repose (θ) was then calculated, employing the following formula (Equation (3)):

(3) $$\tan\left(\theta \right)=\frac{2H}{D}$$

#### 2.4.3. Bulk and Taped Density

A 50 mL sample of the mixture (Vb) was gently poured into a 100 mL graduated cylinder without compression, and the weight was recorded (m). The bulk density (ρ_b_) was computed in g/mL using the following formula (Equation (4)):

(4) $$\left(\rho_{b}\right)=\frac{m}{V_{b}}$$

The determination of the tapped density involved mechanically tapping the sample cylinder by lifting and allowing it to drop under its weight at a rate of approximately 200 drops per minute for 3–4 min (approximately 500 taps), and the final tapped volume of the powder was determined (V_t_). The tapped density (ρ_t_) was then calculated using the following formula (Equation (5)).

(5) $$\left(\rho_{t}\right)=\frac{m}{V_{t}}$$

Carr’s compressibility index and the Hausner ratio were also computed using Equation (6) and Equation (7), respectively.

(6) $$Compressibility\;index=\frac{\rho_{t}-\rho_{b}}{\rho_{t}}\times 100$$

(7) $$Hausner\;ratio=\frac{\rho_{t}}{\rho_{b}}$$

### 2.5. Evaluation of Tablets

The physical attributes of the tablets were assessed in accordance with the procedures outlined in the United States Pharmacopeia (USP) [26]. Various parameters such as the granule size distribution, flowability, and compressibility were examined to ensure optimal tablet compression.

#### 2.5.1. Weight Variation

The weight consistency of the prepared tablets was assessed by weighing 20 randomly selected tablets from each formulation using a digital precision balance (METTLER TOLEDO^®^ ME204E- Columbus, OH, USA). Subsequently, the mean weight ± standard deviation (SD) was computed.

#### 2.5.2. Uniformity of Drug Content

Ten tablets were randomly selected from each formulation, and each tablet was individually analyzed using the HPLC method adopted from El-Sherbiny et al. [27]. The established criteria dictate that, for a tablet batch, at least nine out of ten tablets must exhibit drug amounts within the range of 85% to 115% of the labeled claim. If the initial test does not meet these criteria, an additional 30 tablets will be selected, adhering to the USP guidelines. Importantly, no tablet should deviate beyond the range of 75% to 125% [26].

#### 2.5.3. Hardness and Friability

The tablet hardness was measured, employing a hardness tester (Holland^®^ C50, Bracknell, UK), by assessing the breaking force of 10 tablets. To ensure uniform hardness, the test was conducted at the initiation, midpoint, and end of the tablet-manufacturing process. The recorded breaking force was denoted in Newtons (N).

The tablets’ friability was gauged using the (Erweka^®^ TA, Heusenstamm, Germany) friability tester. Initially, 33 tablets (weighing 6.6 g) were weighed (W1) and subsequently placed into the friabilator. Operating at 25 rpm for 4 min, the machine subjected the tablets to mechanical stress. After removal, the tablets were re-weighed (W2). The percentage of friability was computed using Equation (8):

(8) $$Friability\left(\%\right)=\left(\frac{W_{1}-W_{2}}{W_{1}}\right)\times 100$$

#### 2.5.4. Disintegration Time

The disintegration test was carried out in accordance with the immediate-release tablet requirements outlined in the USP. In this test, six tablets from each experiment were placed in a standard USP disintegration apparatus (Erweka^®^ ZT 320 Series, Heusenstamm, Germany) containing 900 mL of distilled water as the immersion fluid, which was adjusted to 37 ± 0.5 °C. The basket rack assembly was allowed to move up and down at a constant frequency until the tablets had fully disintegrated and passed through the mesh. The disintegration time (DT) of each tablet was noted in minutes, and the means ± SD of the six tablets were calculated for each batch.

#### 2.5.5. In Vitro Dissolution

All formulas were tested for the dissolution rate. The USP38-NF33 dissolution procedure was employed to conduct an in vitro drug release assessment for the immediate release dosage forms. The test involved using six tablets from each formula and subjecting them to the dissolution test using USP apparatus II (Erweka^®^ DT 950- Heusenstamm, Germany) with the paddle rotating at 50 rpm and the temperature was kept at 37 ± 0.5 °C. The test was performed in 900 mL of dissolution media at a pH of 6.8 and samples of 2 mL were withdrawn at 10, 15, 30, 45, and 60 min using a 5 mL plastic syringe. The samples were then filtered using a 0.45 μm membrane filter into the HPLC vials. The concentration of LTD was determined using the validated method of analysis. Then, the percentage amount released versus time was estimated and the dissolution profile was constructed.

A pH of 6.8 was chosen for the dissolution medium to simulate the small intestine environment, where Loratadine absorption primarily occurs. Although Loratadine, a weakly basic drug, has higher solubility at a gastric pH (1.2), the small intestine (pH~6.8) is the key site for drug absorption. This choice aligns with regulatory guidelines and is supported by previous studies using pH 6.8 for Loratadine dissolution testing [28,29].

## 3. Results

Various statistical parameters, including *p*-value, R^2^, and adequate precision, were assessed to identify the best-fitting model. Table 3 summarizes the model fitting and statistical analysis. In all proposed models, the predicted R^2^ values were in close agreement with the adjusted R^2^ values (with a difference of less than 0.2), indicating excellent model fitting. Additionally, all models exhibited *p*-values < 0.05, confirming their statistical significance. The adequate precision values, all exceeding 4.0, further demonstrated that the models were reliable for guiding the formulation and process optimization.

As shown in Table 4, the average granular size (D_50_) of the prepared granules ranged from 206.13 ± 0.35 µm to 423.64 ± 0.81 µm. In addition, the bulk density, a critical factor affecting the granule behavior and tablet quality, ranged from 0.413 ± 0.014 g/mL to 0.533 ± 0.110 g/mL, demonstrating a significant improvement in the bulk density of the produced granules with higher TPGS levels. Furthermore, the granule flowability, a key factor affecting the tablet machine performance and the quality of the final product. The flowability of the granules across the nine formulations, listed in Table 4, ranged from 36.21 ± 0.321° to 30.11 ± 0.214°. The observed reduction in the angle of repose indicates enhanced flow properties, which are essential for achieving a uniform die filling during tablet compression. This improvement in flowability contributes to better weight uniformity, reduced tablet defects, and overall improved tablet quality [25].

As shown in Table 5, the weight variation for all formulations fell within the acceptable range specified by the USP. The prepared tablets exhibited weight variations within ±5% of the target weight, with a relative standard deviation (RSD) of less than 2%, indicating good uniformity. These minimal weight variations can be attributed to differences in the bulk density and flowability of the prepared granules [19]. In addition, the content uniformity of the prepared tablets ranged between 96.7% ± 0.3% and 101.3% ± 0.17%, which is well within the USP acceptance criteria of 85–115%, further confirming the improved granule flow properties discussed earlier.

The crushing strength of the tablets, as listed in Table 5, decreased from 69.431 ± 0.211 to 41.286 ± 0.233 N with increasing TPGS levels. This reduction in hardness is consistent with the formation of denser granules. The DT of the tablets varied between 2.35 ± 1.28 and 12.31 ± 0.36 min. Table 5 reflects the influence of formulation factors on the tablet disintegration behavior.

**Figure 1:**
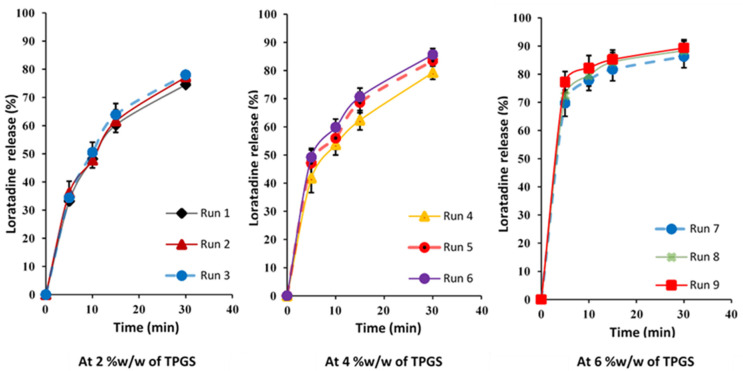
In vitro release profiles of prepared tablets based on 3^2^ Full-Factorial Design.

The in vitro dissolution profile is a key parameter that reflects how the drug is released from the tablet after administration. Several factors, including the tablet composition, granule size, and manufacturing conditions, influence the dissolution performance [30]. The dissolution behavior of each formulation is illustrated in Figure 1, showing that the percentage of the drug released at 30 min ranged from 74.53% ± 1.15% to 91.52% ± 1.98%, as reported in Table 5. Notably, formulations from Runs 4 to 9 demonstrated optimal drug release profiles, achieving an over 80% LTD release within 30 min, meeting the desired dissolution criteria for the immediate-release tablet.

## 4. Discussion

### 4.1. Influence of Key Variables on Average Granules Size (D_50_)

The following mathematical models (Equation (9)) were used to determine the impact of independent factors on the d_50_.

(9) $$D_{50}\left(\mu m\right)=339.49+90.82X_{1}+20.91X_{2}-8.53X_{1}X_{2}-17.28{X_{1}}^{2}-0.577{X_{2}}^{2}$$

The ANOVA analysis shown in Table 6 demonstrated that both TPGS and SSG had a significant effect on the granule size (*p* < 0.0001 for TPGS and *p* = 0.0003 for SSG). The regression equation (Equation (9)) revealed a positive relationship between the major terms and granule size. Specifically, increasing the levels of TPGS and SSG significantly increased the granule size (+90.82 and +20.91, respectively). TPGS had a more pronounced effect (+90.82) compared to SSG (+20.91). The sum of squares values further confirmed that TPGS had a predominant impact on the granule size (49,495.08 for TPGS vs. 2623.37 for SSG). Additionally, the interaction terms X_1_X_2_ (*p* = 0.432) and the quadratic term X_12_ (*p* = 0.0003) had also a significant impact on the size of the granules.

**Figure 2:**
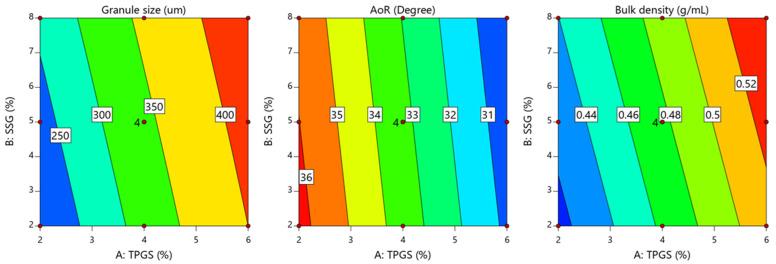
Contour plot showing the influence of TPGS (X1) and SSG (X2) on average granule size, flowability, and bulk density.

These findings align with previously published data by Pandey et al., where TPGS formulations demonstrated significantly higher granule mean diameter values compared to conventional formulations using hydroxypropyl cellulose as a binder [31]. Pandey et al. reported that TPGS-containing formulations achieved superior granule growth and exhibited sufficient mechanical strength to withstand the fluid-bed drying process [31]. This confirms the effectiveness of TPGS as a binder, particularly when processed using high-shear wet granulation, eliminating the need for a conventional binder. The contour plot in Figure 2 supports this observation, showing that when both TPGS and SSG concentrations were increased, the granule size reached its maximum. The granule size increase observed in this study further supports the hypothesis that TPGS facilitates the coalescence of powder particles during wet granulation. The binding action of TPGS enhances the adherence and agglomeration of particles, resulting in larger and more robust granules. When the concentration of TPGS increased from 2% to 6% *w*/*w*, the granule size doubled (Table 4 and Figure 2), demonstrating the significant role of TPGS in the granule formation.

This finding is consistent with reports by Pandey et al., indicating that TPGS not only acts as a solubilizing agent, but also as an effective binder [31]. The binding capability of TPGS at concentrations between 2% and 6% *w*/*w* contributes to the granule robustness, suggesting that such formulations are capable of withstanding the stresses of downstream processing without compromising the granule integrity. These properties are critical in the development of solid dosage forms, where the granule size and strength play a key role in ensuring the uniformity, compressibility, and dissolution performance [4,11].

### 4.2. Influence of Key Variables on Granules’ Bulk Density

The following mathematical models (Equation (10)) were used to determine the effect of separate formulation factors on the granules’ bulk density.

(10) $$Bulk\;density\left(g/mL\right)=0.4762+0.0495X_{1}+0.013X_{2}$$

The ANOVA analysis shown in Table 6 demonstrated that the level of TPGS and SSG had a significant effect on the bulk density of the prepared granules (*p* < 0.0001 for TPGS and *p* = 0.05 for SSG). Equation (10) demonstrates that an increase in the concentration of TPGS and SSG led to a greater increase in the bulk density of the produced granules, as shown by the positive sign of the coefficient estimates (+0.0495 for TPGS and +0.013 for SSG). Furthermore, Figure 2 highlights the pronounced effect of TPGS on the granules’ bulk density in a positive direction. This observation suggests that higher levels of TPGS promote granule consolidation, resulting in denser granules. This finding is consistent with the report by Sotthivirat et al., which also noted that the presence of TPGS enhances the granule compactness and density [11].

The results of this study demonstrate a positive correlation between the granule size and bulk density. As the granule size increased, the bulk density also showed a significant rise. This relationship can be explained by the reduction in inter-particle void spaces as the granules grow larger and become more consolidated. Larger granules tend to pack more efficiently, reducing void spaces and increasing the bulk density [32].

In this study, the contour plots in Figure 2 indicate that increasing the levels of TPGS led to a simultaneous increase in both the granule size and bulk density. This suggests that TPGS not only acted as a binder to promote granule formation, but also contributed to granule consolidation. The coalescence of powder particles facilitated by TPGS reduced the porosity of the granules, making them denser. The findings align with previous reports by Pandey et al. [31] and Sotthivirat et al. [11], who also observed that formulations containing TPGS showed larger granule sizes and higher bulk densities compared to conventional formulations. These studies confirmed that TPGS enhances both the size and compactness of granules, leading to improved flow properties and compressibility during tablet manufacturing. Therefore, the correlation between the granule size and bulk density observed in this study further validates the role of TPGS as an effective binder in the wet granulation process.

### 4.3. Influence of Key Variables on Granules’ Flowability

The mathematical model generated by a regression analysis of the granules’ flowability is described by Equation (11).

(11) $$Angle\;of\;repose\left(degree\right)=33.26-2.76X_{1}-0.300X_{2}$$

The ANOVA results shown in Table 6 indicate that both TPGS and SSG significantly influenced the granule flowability, with *p*-values of <0.0001 for TPGS and 0.0291 for SSG. However, the sum of the squares’ values highlights that TPGS had a more pronounced effect on the granule flowability (45.71 for TPGS vs. 0.54 for SSG), suggesting that TPGS plays a dominant role in enhancing the flow characteristics of the granules. The regression coefficients in Equation (11) further confirm the positive influence of both TPGS and SSG on the granule flowability, as indicated by their negative signs (−2.76 for TPGS and −0.300 for SSG), which reflect a reduction in the angle of repose and the improved granule flow. The contour plot in Figure 2 illustrates that a simultaneous increase in the TPGS and SSG levels resulted in the maximum granule flowability. This improvement in flowability can be attributed to the enhanced granule size and reduced interparticle friction, which are critical factors in achieving optimal flow properties [33]. Notably, granules exhibited the best flowability when both TPGS and SSG concentrations were increased, underscoring the synergistic effect of these excipients in improving bulk properties.

The findings of this study align with the study by Sotthivirat et al., which reported that TPGS-based high-shear wet granulation (HSWG) formulations provide several advantages, particularly in improving flow properties and enhancing process robustness. Sotthivirat’s findings underscore that TPGS formulations are well-suited for poorly water-soluble drugs with inherently challenging flow characteristics [11]. Similarly, Pandey et al. demonstrated that TPGS-based formulations processed through HSWG exhibited robust granulation properties and improved flow characteristics [31]. Additionally, Sotthivirat et al. highlighted that HSWG for TPGS-based formulations facilitates more robust downstream processing by mitigating issues such as cohesivity and static charge-induced agglomeration, which are commonly observed in formulations of poorly water-soluble drugs [4]. This aligns with our findings that the incorporation of TPGS leads to larger granule sizes and improved flow properties, reducing the likelihood of cohesive behavior during processing.

### 4.4. Influence of Key Variables on Tablet Strength

The mathematical model generated by regression analysis of tablet-crushing strength is described by Equation (12).

(12) $$\begin{array}{c} Crushing\;strength\left(N\right)\\ \;\;\;\;\;\;\;=68.647-1.2X_{1}-0.07X_{2}-0.25\;X_{1}X_{2}-1.44\;{X_{1}}^{2}\\ \;\;\;\;\;\;\;\;-0.058\;{X_{2}}^{2} \end{array}$$

**Figure 3:**
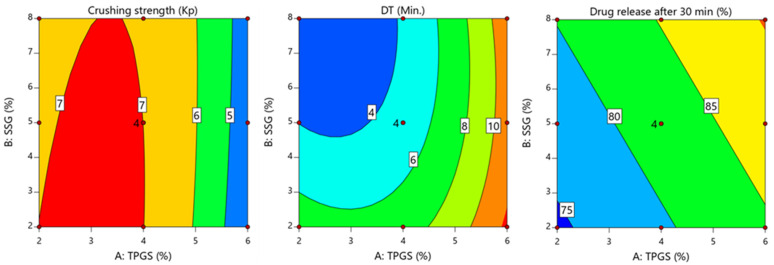
Contour plot showing the influence of TPGS (X_1_) and SSG (X_2_) on crushing strength, DT, and drug release.

The ANOVA results in Table 7 revealed that the tablets’ crushing strength was significantly affected by TPGS (*p* < 0.0001 for TPGS and *p* = 0.267 for SSG). The negative regression coefficient for TPGS (−1.2) indicates that the increasing TPGS concentration led to a decrease in the crushing strength. This observation aligns with the contour plot in Figure 3, which shows a reduction in the crushing strength at higher TPGS levels.

The quadratic model further revealed significant nonlinear relationships for TPGS^2^ (*p* < 0.0001) and the interaction term TPGS × SSG (*p* = 0.013). The negative coefficient for TPGS^2^ (−1.44) indicates that the effect of TPGS on the crushing strength diminishes at higher levels, leading to a plateau or further decrease in tablet hardness. This is likely due to the lubricating and plasticizing properties of TPGS, which reduce interparticle friction and compressibility at higher concentrations [8]. The positive coefficient for the interaction term TPGS × SSG (0.25) suggests a synergistic effect between TPGS and SSG at moderate levels, enhancing the tablet strength. However, the quadratic term for SSG^2^ was not significant (*p* = 0.5294), indicating that SSG levels have a linear relationship with the crushing strength within the studied range.

The reduction in the crushing strength observed with increasing TPGS levels can be attributed to the increase in the bulk density and decrease in the granule porosity. The granule porosity plays a critical role in determining a tablet’s compaction behavior. The higher porosity in granules provides more bonding surface area and allows for greater particle deformation and fragmentation during the compression process, which ultimately leads to stronger tablet structures [34]. However, when TPGS is incorporated at higher levels, it promotes the formation of denser granules with reduced porosity. These dense granules have lower compressibility, meaning they undergo less deformation during tablet compression, resulting in weaker inter-particle bonds. Additionally, the loss of porosity reduces the number of potential bonding sites between particles, thereby impairing the mechanical strength of the resulting tablets [31]. Moreover, TPGS, being an amphiphilic surfactant, can act as a lubricant-like substance, reducing the inter-particle friction between granules during compression. This lubricating effect further decreases the tablet hardness, as it limits the degree of particle bonding during the compaction process [8].

While this may raise concerns about the tablet stability, handling, and packaging, the observed hardness values (41.286 ± 0.233 − 69.431 ± 0.211 N) remain within acceptable limits for immediate-release tablets, ensuring adequate mechanical strength for handling and packaging. Furthermore, the use of appropriate packaging materials, such as blister packs or bottles with desiccants, can mitigate potential stability issues caused by lower hardness. These findings suggest that while TPGS levels influence the tablet hardness, the formulation remains robust and suitable for commercial production.

This effect is consistent with previously published work by Sotthivirat et al., who reported that TPGS levels ranging from 2.2 to 6.2 wt% in tablet formulations resulted in decreased tablet strength. The study noted that this reduction in strength was linked to the higher bulk densities of granules formed with elevated TPGS concentrations, which promote granule wetting, nucleation, consolidation, and growth. These denser granules, although beneficial for flow and uniformity, tend to produce tablets with a reduced crushing strength due to their compact structure [4]. Similarly, a study by Pandey et al. demonstrated that increasing TPGS levels during the granulation process led to denser granules, which, while improving the flowability and handling, negatively impacted the crushing strength of the final tablets [8,31].

Furthermore, the friability of the prepared tablets in our study ranged from 0.02 ± 0.06% to 0.17 ± 0.05%, which is well within the acceptable range according to USP standards. The low friability values suggest that despite the reduction in the crushing strength, the tablets remain robust enough to withstand handling and transportation. This balance between an adequate mechanical strength and acceptable friability underscores the suitability of TPGS as a multifunctional excipient, especially for poorly water-soluble drugs requiring enhanced dissolution and flow properties.

### 4.5. Influence of Key Variables on Disintegration Time

These mathematical models, represented by Equation (13), accounted for the impact of outside factors on the disintegration time of the finished tablets.

(13) $$DT\left(min.\right)=4.87+3.53X_{1}-1.5X_{2}+0.975X_{1}X_{2}+2.62{X_{1}}^{2}+0.856{X_{2}}^{2}$$

The ANOVA results in Table 7 indicate that both factors had significant effects on the disintegration time, with TPGS (*p* < 0.0001) having a more pronounced influence than SSG (*p* = 0.0002). The sum of squares values (74.69 for TPGS vs. 13.44 for SSG) further confirm that TPGS had a dominant role in delaying disintegration. The regression coefficients indicate that increasing the concentration of TPGS significantly delays the disintegration time, whereas SSG accelerates it.

The quadratic model revealed significant nonlinear relationships for TPGS^2^ (*p* < 0.0001) and SSG^2^ (*p* = 0.0237), as well as a significant interaction term TPGS × SSG (*p* = 0.0057). The positive coefficient for TPGS^2^ (2.62) suggests that higher TPGS levels significantly prolong the disintegration time, likely due to granule densification and reduced porosity. The positive coefficient for SSG^2^ (0.8563) indicates that the effect of SSG on the disintegration time increases at higher levels, potentially due to its superdisintegrant properties becoming more pronounced. The interaction term TPGS × SSG (0.975) shows a synergistic effect, where the combination of TPGS and SSG at moderate levels further influences the disintegration time. These findings highlight the importance of optimizing TPGS and SSG levels to balance the tablet integrity and disintegration performance.

As shown in Figure 3, the disintegration time reached its peak at higher TPGS levels, particularly on the right side of the contour plot. This delay can be attributed to granule densification caused by higher TPGS concentrations, which reduces the porosity and slows water penetration into the tablet matrix. This aligns with the findings of Sotthivirat et al., who reported that increasing TPGS levels decreases the tablet compactability, but slightly delays disintegration due to its effect on the granule structure and bulk density [4]. The enhanced granule densification at higher TPGS levels forms a more compact and less porous tablet matrix, thus reducing the rate at which water can enter and initiate disintegration.

Additionally, Pandey et al. found that adding extra-granular Prosolv^®^ (silicified microcrystalline cellulose) to formulations containing TPGS improved the tablet tensile strength and reduced the disintegration time, particularly at higher TPGS levels [31]. This suggests that incorporating porous and highly absorbent excipients can counteract the delaying effect of TPGS on disintegration. However, in the current study, the focus was on the impact of TPGS and SSG alone, without additional extra-granular excipients, which may explain the observed delay in the disintegration time.

While TPGS delays disintegration due to its hydrophobic nature and granule densification, SSG acts as a super-disintegrant, promoting the water uptake and swelling, which facilitates faster tablet break-up. This balance between the binder and disintegrant levels is crucial for optimizing the disintegration time of tablets. Further studies could explore the use of alternative superdisintegrants, such as croscarmellose sodium or crospovidone, or the inclusion of absorbent excipients to optimize both dissolution and disintegration properties.

### 4.6. Influence of Key Variables on Tablet Dissolution

The following mathematical models of Equation (14) addressed the effect of formulation factors on the dissolution of manufactured tablets. This observation aligns with the contour plot in Figure 3.

(14) $$Percent\;release\;at\;30min.\left(\%\right)=82.26+5.06X_{1}+2.98X_{2}$$

The ANOVA results presented in Table 7 demonstrate that both TPGS and SSG significantly influenced the dissolution behavior of the manufactured tablets, with *p*-values of <0.0001 and <0.0003, respectively. The positive coefficients in Equation (14) indicate that increasing the levels of both factors enhances the percentage of drug release at 30 min. Among these factors, TPGS had a more pronounced effect on dissolution, as evidenced by the higher sum of squares value (153.42 for TPGS compared to 53.34 for SSG). This indicates that TPGS is the dominant factor in improving the dissolution profile. As illustrated in Figure 3, the percent drug release at 30 min increased with the simultaneous increase in both TPGS and SSG levels. However, the influence of TPGS was more substantial, underscoring its critical role in enhancing the dissolution of poorly soluble LTD. The superior influence of TPGS on tablet dissolution can be attributed to its surfactant properties, which play a critical role in improving the solubility and release of poorly water-soluble drugs like LTD. The TPGS is a non-ionic surfactant with an HLB value of 13.2 and a low critical micelle concentration of 0.02% *w*/*w*, making it highly effective in reducing interfacial tension between the drug particles and the dissolution medium [35]. By lowering the interfacial tension, TPGS enhances the wetting of drug particles and facilitates the formation of micelles, which encapsulate the drug and improve its dispersion in the dissolution medium [36]. The amphiphilic nature of TPGS, characterized by its hydrophilic polar head and lipophilic alkyl tail, allows it to adsorb onto the surface of drug particles, creating localized areas with a high concentration of surfactants. This facilitates the solubilization of the poorly soluble LTD, thereby increasing its release into the bulk solution [36,37]. This mechanism aligns with the findings reported by Sotthivirat et al., who demonstrated that TPGS significantly improved the dissolution of hydrophobic drugs by enhancing their wetting, solubilization, and dispersion properties [4]. Furthermore, the ability of TPGS to form micelles at low concentrations promotes the incorporation of the drug within micellar structures, thereby increasing the surface area available for dissolution. Additionally, TPGS can inhibit drug recrystallization, maintaining the drug in an amorphous form, which further contributes to improved dissolution and absorption [36].

### 4.7. Lack-of-Fit Test

The lack-of-fit test is a widely employed statistical method for evaluating the adequacy of a mathematical model by comparing the residual error with the pure error. This test is crucial for determining whether the proposed model accurately represents the experimental data or if a significant portion of variability remains unexplained. The test returns F values, which are then compared to the corresponding tabulated F values to assess the model’s validity [38].

As shown in Table 8, the lack-of-fit test results indicated that there was no statistically significant mismatch between the observed data and the predicted values, as the calculated F values were larger than the tabulated F values for all responses. This confirms that the proposed models adequately fit the data, enhancing confidence in the accuracy of the regression equations and the reliability of the predictions made using these models.

As shown in Table 8, the lack-of-fit test results indicated that there was no statistically significant mismatch between the observed data and the predicted values for most responses, as the calculated F values were larger than the tabulated F values. However, for drug release, the lack-of-fit test was significant (*p* < 0.05), indicating that the model did not fully capture the variability in the drug release data. This suggests that additional factors or higher-order terms may be needed to improve the model’s fit for drug release. Despite this, the proposed models adequately fit the data for the other responses, enhancing confidence in the accuracy of the regression equations and the reliability of the predictions made using these models.

### 4.8. Optimization and Validation of Design

**Figure 4:**
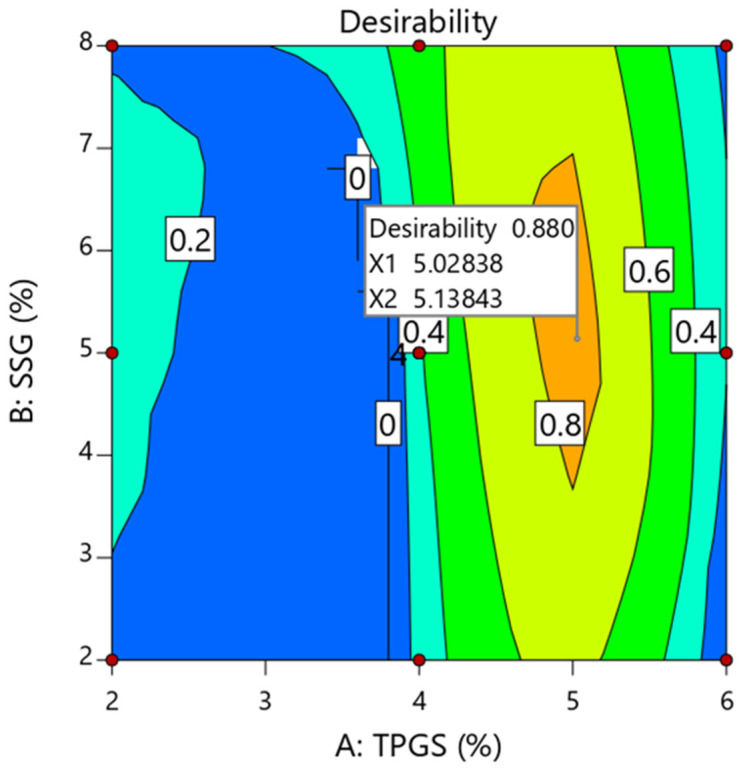
Formulation optimization using the desirability function.

The formulation may be optimized by defining objectives for each answer and then applying the global desirability function concurrently. The crushing strength, disintegration duration, and drug release after 30 min were all set to 6 KP, 5 min, and 85%, as indicated in Table 9. Based on these parameters, the desirability plot was constructed with a high D value of 0.872 (Figure 4). Furthermore, optimization using the desirability function revealed that TPGS and SSG at high (5.02% *w*/*w*) and moderate (5.13% *w*/*w*) levels, respectively, were the best for producing tablets with the desired quality attributes. Furthermore, after 30 min, the optimized formulation demonstrated a crushing strength of 5.72 ± 0.32 KP, a disintegration time of 7.11 ± 1.08, and a percent release of 86.21 ± 1.61%, all of which match the USP acceptance standards for immediate-release tablets. The prediction error was determined using anticipated and observed values, as shown in Table 10, and the results were found to be within the allowed range (±5%), verifying the design’s precision. In addition, the prediction error of −1.42% indicates that the predicted value was 1.42% lower than the observed value, reflecting a slight underestimation by the model.

### 4.9. Scale-Up Considerations and Commercial Viability

The findings of this study demonstrate the potential of TPGS and SSG in enhancing the dissolution properties of LTD tablets. Based on a prior study, TPGS-based formulations provide a wide operating window for high-shear wet granulation processing, with a minimal impact on the product performance across different granulators [11]. This suggests greater flexibility and potential success for scale-up and commercial development. However, certain challenges, such as ensuring consistent granule size distribution and flow properties during large-scale wet granulation, as well as maintaining the tablet integrity and drug release performance during high-speed compaction, must be addressed. By leveraging established pharmaceutical manufacturing technologies and process optimization strategies, these challenges can be effectively managed, making the formulation suitable for commercial production.

The practical implications of these findings extend beyond LTD to other BCS Class II drugs, which often face similar challenges related to poor solubility and bioavailability. The optimized levels of TPGS and SSG identified in this study can be applied to enhance the dissolution of other poorly water-soluble drugs by improving the wetting, reducing interfacial tension, and ensuring rapid disintegration. Additionally, the use of TPGS as a solubilizing agent and SSG as a superdisintegrant can be extended to other formulations requiring immediate-release properties. This approach not only improves drug release, but also ensures robustness in manufacturing, making it a versatile strategy for formulating BCS Class II drugs.

## 5. Conclusions

The present study successfully demonstrated the application of the DoE approach to optimize the formulation of LTD tablets with enhanced dissolution profiles. The use of a 3^2^ full-factorial design allowed for a systematic evaluation of the effects of two critical formulation variables, TPGS and SSG, on multiple dependent responses, including the granule flowability, tablet mechanical strength, DT, and drug release. The DoE approach provided a robust platform for understanding the interactions between these variables and identifying the optimal formulation conditions, minimizing the need for extensive trial-and-error experimentation.

A regression analysis confirmed that both TPGS and SSG levels significantly (*p* ≤ 0.05) influenced the granule flowability, tablet mechanical strength, DT, and dissolution behavior, with TPGS having the most pronounced effect across all responses. The optimization process using the desirability function identified that the optimal formulation required high levels of TPGS (5% *w*/*w*) and moderate levels of SSG (5% *w*/*w*) to achieve tablets with the desired quality attributes. The optimized LTD tablets met the desired quality specifications, demonstrating a robust performance in terms of the crushing strength, DT, and percent drug release at 30 min.

In conclusion, the inclusion of TPGS in the granulation process proved to be a key factor in enhancing the dissolution of poorly water-soluble drugs. The amphiphilic nature of TPGS, combined with its ability to reduce interfacial tension and improve the wetting of drug particles, contributed to the superior dissolution behavior. The improved dissolution is expected to enhance the bioavailability and therapeutic efficacy as a faster and more complete drug release in the gastrointestinal tract can lead to higher plasma concentrations and better clinical outcomes.

This study highlights the potential of TPGS as a versatile excipient in the formulation of poorly soluble drugs to enhance bioavailability and ensure formulation robustness in pharmaceutical manufacturing. The findings also underscore the robustness and scalability of the formulation, making it a promising candidate for industrial application. The use of TPGS in high-shear wet granulation has been shown to provide a wide operating window, with minimal impact on the product performance across different granulators, as supported by prior studies. While challenges such as ensuring a consistent granule size distribution and flow properties during scale-up may arise, these can be effectively managed through established pharmaceutical manufacturing technologies and process optimization strategies. These results underscore the industrial applicability of the formulation, paving the way for further development and commercialization.

## Figures and Tables

**Table 1:** A full factorial experimental design with 3^2^ runs.

Run	TPGS (% *w*/*w*)	SSG (% *w*/*w*)
1	2	2
2	2	5
3	2	8
4	4	2
5	4	5
5 *	4	5
5 *	4	5
5 *	4	5
6	4	8
7	6	2
8	6	5
9	6	8

* Repeated center point.

**Table 2:** Formulation used in the preparation of loratadine tablets.

Ingredients	Function	% *w*/*w*
Loratadine	Drug	5
TPGS	Surfactant	2–6
Sodium starch glycolate	Superdisintegrant	2–8
* Microcrystalline cellulose PH-102	Filler	Up to 100
* Lactose monohydrate	Filler	Up to 100
Magnesium stearate	Hydrophobic lubricant	1
Total	-	100

* Microcrystalline cellulose PH-102/ Lactose monohydrate added up to 100% with 1:1 ratio.

**Table 3:** A synopsis of data fitting and statistical analysis.

Responses	Suggested Model	*p*-Value	R^2^	Adjusted R^2^	Predicted R^2^	Adequate Precision
Y_1_: * D_50_	Quadratic	<0.0001	0.9950	0.9908	0.9759	47.31
Y_2_: Bulk density	Linear	<0.0001	0.8979	0.8753	0.8172	17.74
Y_3_: Angle of repose	Linear	<0.0001	0.9846	0.9812	0.9779	43.16
Y_4_: Crushing strength	Quadratic	<0.0001	0.9920	0.9853	0.9336	28.52
Y_5_: Disintegration time	Quadratic	<0.0001	0.9892	0.9803	0.9715	30.58
Y_6_: Percent release after 30 min	Linear	<0.0001	0.9331	0.9183	0.8529	25.05

* D_50_ is the average granular size.

**Table 4:** Measured response results of prepared granules (mean ± SD).

Formula	Mean Granule Size (µm ± SD)	Bulk Density (g/mL ± SD)	Tapped Density (g/mL ± SD)	Angle of Repose (Degree ± SD)	Carr’s Index	Hausner’s Ratio
1	206.13 ± 0.35	0.413 ± 0.014	0.510 ± 0.005	36.21 ± 0.321	19	1.23
2	224.71 ± 0.32	0.431 ± 0.006	0.502 ± 0.009	35.89 ± 0.423	14.1	1.16
3	262.15 ± 0.21	0.452 ± 0.032	0.518 ± 0.019	35.57 ± 0.127	12.7	1.14
4	311.56 ± 0.26	0.463 ± 0.034	0.542 ± 0.007	33.46 ± 0.615	14.5	1.17
5	331.25 ± 0.21	0.461 ± 0.007	0.533 ± 0.015	33.54 ± 0.247	13.5	1.15
5 *	346.11 ± 0.65	0.458 ± 0.033	0.521 ± 0.0024	33.11 ± 0.442	12.1	1.13
5 *	344.21 ± 0.24	0.456 ± 0.008	0.513 ± 0.017	33.78 ± 0.361	11.1	1.12
5 *	343.54 ± 0.32	0.486 ± 0.047	0.561 ± 0.031	33.61 ± 0.431	13.3	1.15
6	359.11 ± 0.45	0.501 ± 0.019	0.568 ± 0.003	32.84 ± 0.392	11.8	1.13
7	401.75 ± 0.64	0.532 ± 0.020	0.587 ± 0.012	30.77 ± 0.331	9.3	1.10
8	412.55 ± 0.25	0.528 ± 0.022	0.579 ± 0.043	30.11 ± 0.214	8.8	1.09
9	423.64 ± 0.81	0.533 ± 0.110	0.591 ± 0.027	30.23 ± 0.325	9.8	1.11

* Repeated center point.

**Table 5:** Measured response results of prepared tablets (mean ± SD).

Run	Weight (mg ± SD)	Drug Content (% ± SD)	Crushing Strength (N ± SD)	Friability (% ± SD)	Disintegration Time (S ± SD)	%Release at 30 min (% ± SD)
1	200.6 ± 0.951	100.12 ± 1.31	68.352 ± 0.215	0.09 ± 0.03	7.11 ± 1.13	74.53 ± 1.15
2	201.03 ± 0.624	96.70 ± 0.13	68.058 ± 0.133	0.02 ± 0.06	4.15 ± 1.33	77.24 ± 1.14
3	200.59 ± 0.943	101.23 ± 1.12	61.095 ± 0.139	0.03 ± 0.02	2.35 ± 1.28	78.04 ± 1.13
4	201.0 5 ± 0.701	100.66 ± 1.6	68.254 ± 0.235	0.03 ± 0.01	7.5 ± 1.63	79.25 ± 2.36
5	200.96 ± 0.829	98.3 ± 1.61	69.431 ± 0.211	0.17 ± 0.05	5.38 ± 1.61	83.47 ± 1.82
5 *	200.81 ± 0.853	101.54 ± 0.88	68.745 ± 0.210	0.04 ± 0.03	4.25 ± 1.32	82.79 ± 2.41
5 *	200.47 ± 0.683	100.88 ± 1.63	67.568 ± 0.237	0.09 ± 0.02	5.33 ± 2.18	83.14 ± 2.84
5 *	201.08 ± 0.755	98.76 ± 2.01	67.764 ± 0.209	0.06 ± 0.01	4.35 ± 0.57	82.91 ± 1.21
6	200.77 ± 1.06	100.93 ± 0.76	68.745 ± 0.342	0.15 ± 0.03	4.14 ± 0.81	85.63 ± 2.17
7	200.79 ± 0.871	100.4 ± 0.8	41.286 ± 0.233	0.08 ± 0.01	12.31 ± 0.36	83.52 ± 2.33
8	200.84 ± 0.739	101.3 ± 1.7	41.776 ± 0.257	0.16 ± 0.03	11.02 ± 0.84	85.11 ± 3.11
9	200.61 ± 0.828	100.1 ± 1.5	43.836 ± 0.353	0.12 ± 0.01	11.45 ± 0.68	91.52 ± 1.98

* Repeated center point.

**Table 6:** ANOVA analysis of granules’ measured responses.

Variables	Coefficient Estimate	Sum of Squares	Standard Error	* F-Value	*p*-Value
**Granule size (Quadratic model)**					
Intercept	339.49	-	2.05	-	-
X_1_	90.82	49,495.08	2.73	1109.18	<0.0001
X_2_	20.91	2623.37	2.73	58.79	0.0003
X_1_X_2_	–8.53	291.21	3.34	6.53	0.0432
X_1_^2^	–17.28	796.49	–17.28	17.85	0.0055
X_2_^2^	–0.5775	0.8893	–0.5775	0.0199	0.8924
**Granules bulk density (Linear model)**					
Intercept	0.4762	–	0.0041	–	–
X_1_	0.0495	0.0147	0.0058	74.08	<0.0001
X_2_	0.013	0.0010	0.0058	5.11	0.0501
**Granule flowability (Linear model)**					
Intercept	33.26	-	0.0819	-	-
X_1_	–2.76	45.71	0.1158	568.48	<0.0001
X_2_	–0.30	0.54	0.1158	6.72	0.0291

X_1_ and X_2_ are TPGS and SSG levels, respectively. * F-value: Fisher criterion, used to test the significance of factors in the ANOVA analysis. A higher F-value indicates a greater significance of the factor.

**Table 7:** ANOVA analysis of prepared tablets measured responses.

Variables	Coefficient Estimate	Sum of Squares	Standard Error	* F-Value	*p*-Value
**Crushing strength (Quadratic model)**					
Intercept	7.0	-	0.06	-	-
X_1_	–1.2	8.64	0.05	417.91	<0.0001
X_2_	–0.071	0.03	0.05	1.49	0.267
X_1_X_2_	0.25	0.25	0.07	12.09	0.013
X_1_^2^	–1.44	5.56	0.0881	268.86	<0.0001
X_2_^2^	–0.058	0.0092	0.0881	0.4452	0.529
**Disintegration time (Quadratic model)**					
Intercept	4.87	-	0.212	-	-
X_1_	3.53	74.69	0.189	345.88	<0.0001
X_2_	–1.5	13.44	0.189	62.23	0.0002
X_1_ X_2_	0.975	3.8	0.232	17.61	0.0057
X_1_^2^	2.62	18.32	0.2846	84.84	<0.0001
X_2_^2^	0.856	1.96	0.2846	9.05	0.0237
**Percent release at 30 min (Linear model)**					
Intercept	82.26	-	0.37	-	-
X_1_	5.06	153.42	0.52	93.19	<0.0001
X_2_	2.98	53.34	0.52	32.4	0.0003

X_1_ and X_2_ are TPGS and SSG levels, respectively, X_1_X_2_ is the effect of interaction. * F-value: Fisher criterion, used to test the significance of factors in the ANOVA analysis. A higher F-value indicates a greater significance of the factor.

**Table 8:** Lack-of-fit test of measured responses.

Response	* F-Value	*p*-Value	Comment
d50	0.945	0.517	Not significant
bulk density	1.02	0.537	Not significant
Flowability	0.984	0.551	Not significant
Crushing strength	4.24	0.133	Not significant
Disintegration time	0.157	0.918	Not significant
Percent release at 30 min	27.07	0.01	Significant

* F-value: Fisher criterion, used to test the significance of factors in the ANOVA analysis. A higher F-value indicates a greater significance of the factor.

**Table 9:** The constraints adopted for developed design space.

Variables	Target	Range	Weight	Importance Coefficient
Input				
TPGS	In range	2–6% *w*/*w*	1	NA
SSG	In range	2–8% *w*/*w*	1	NA
Output				
Crushing strength (N)	58.840	41.286–69.431		+++
Disintegration time (Min)	5	2.35–12.31	1	+++
Percent release at 30 min (%)	85	74.53–91.52	1	+++

**Table 10:** Predicted and experimental values of optimized run with their relative errors.

Variables	Value		
TPGS (2–6% *w*/*w*)	5.02		
SSG (2–8% *w*/*w*)	5.13		
Overall desirability = 0.880			
Responses	Predicted values	Experimental values *	Prediction error (%)
Crushing strength (N)	58.84	56.094 ± 0.32	4.66
Disintegration time (Min.)	7.33	7.11 ± 1.08	3.001
Percent release at 30 min (%)	85.0	86.21 ± 1.61	–1.42

* Experimental (actual) values are presented as mean ± SD.

## Data Availability

The research data used in the preparation of the manuscript will be available upon request.
